# A case of extreme hyponatremia without neurologic symptoms

**DOI:** 10.1002/ccr3.2383

**Published:** 2019-08-20

**Authors:** Na Zhou, Chang Yang

**Affiliations:** ^1^ Kidney Research Clinic University of Utah School of Medicine Salt Lake City Utah; ^2^ Apogee Hospital Medicine Passavant Area Hospital Jacksonville Illinois

**Keywords:** adrenal insufficiency (AI), hyponatremia, hypothyroidism, trimethoprim‐sulfamethoxazole (TMP‐SMX)

## Abstract

Our case report highlights that profound hyponatremia with sodium level 101 mmol/L could have no CNS symptoms, and drugs and endocrine disorders are relatively common causes and should be considered in the differential diagnosis of hyponatremia. Standard dose trimethoprim‐sulfamethoxazole‐induced hyponatremia is rare but still worth close attention in clinical practice.

## 1 INTRODUCTION

Hyponatremia is one of the most common electrolyte abnormalities encountered in clinical practice, occurring in 15%‐30% of hospitalized patients and has been associated with considerable morbidity and mortality.^1^ But unfortunately, this common disorder remains incompletely understood, because it can be caused by multiple etiologies with different pathophysiological mechanisms, and it is usually associated with a variety of underlying diseases, making the diagnosis and treatment complicated and challenging in some cases. Medications are a common cause of electrolyte abnormalities including hyponatremia, such as high‐dose trimethoprim‐sulfamethoxazole (TMP‐SMX)‐induced electrolyte abnormality has been widely observed. But reports about standard dose TMP‐SMX‐induced hyponatremia are rare. Endocrine disorders including adrenal insufficiency (AI) and hypothyroidism‐induced hyponatremia should not be ignored either and should be considered in the differential diagnosis.

## 2 CASE REPORT

### 2.1 Patient information

A 54‐year‐old Caucasian female presented to the emergency department with nausea, vomiting, and fatigue for 5 days. She had visited her primary care physician for these symptoms and was given ondansetron and normal saline infusion without obvious improvement. With further questioning, the patient stated that she had felt poorly for at least 1 month, including poor appetite, fatigue, and unintentional weight loss. She had occasional vague abdominal pain which usually resolved spontaneously. She stated that her skin recently became very tan which she attributed to be in Arizona a month prior. The patient has a 17‐year history of hypothyroidism. She was initially treated with levothyroxine, but she had decided to switch to animal thyroid extract several months prior. The patient started taking TMP‐SMX 160 mg b.i.d. for sinus infection 1 week prior.

### 2.2 Physical exam

On admission, her orthostatic was positive: supine BP 131/80 mm Hg and HR 81/min; standing BP 107/67 mm Hg and HR 96/min. Otherwise her vital signs were within normal range. The patient was ill appearing, generally dehydrated with dry skin and mucosa. Capillary refill time was 3 seconds. The abdomen was not distended, normal bowel sounds, mild tenderness to palpation in the epigastric area, and no rebound tenderness or guarding. The remainder of the clinical examinations including heart and lungs were normal. Patient was alert and oriented, and no focal neurologic deficits observed. Further neurologic assessment by several specialists did not reveal any pathological signs.

### 2.3 Diagnostic assessment

Initial laboratory tests showed the following: WBC 10.2 k/µL, RBC 4.95 k/µL, hemoglobin 15.1 g/dL, hematocrit 47%, sodium 101 mmol/L, chloride 73 mmol/L, potassium 5.2 mmol/L, CO2 16 mmol/L, AG 11, BUN 20 mg/dL, Cr 0.8 mg/dL, and eGFR > 60 mL/min/1.73 m^2^. The rest of the biochemical analysis including liver function, calcium, phosphorus, glucose, total protein albumin, lipid panel, and globulin were within normal range.

**Figure 1:**
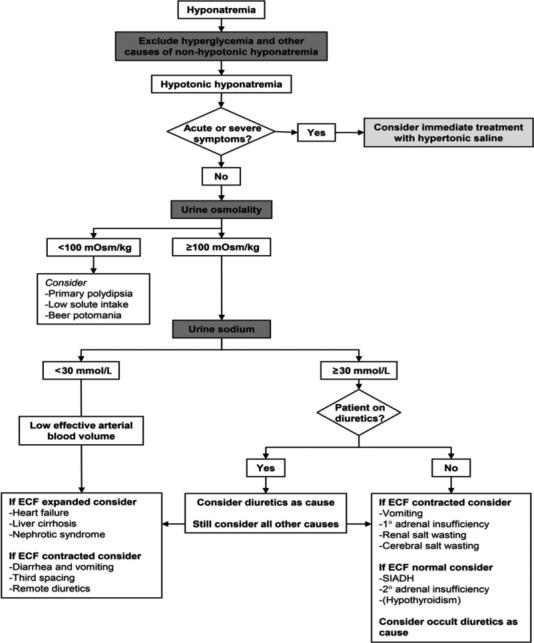
Diagnostic algorithm for hyponatremia from the European guideline

With patient's extremely low serum sodium, our attention focused on the investigation of the cause. Further tests showed plasma osmolality 225 mOsm/kg (275‐295 mOsm/kg), sodium in urine sample was 128 mmol/L, and urine osmolality 128 mOsm/kg. Since hyperglycemia and other causes of nonhypotonic hyponatremia were excluded, our patient had hypotonic hypovolemic hyponatremia. Given increased urine osmolality, elevated urine sodium, and no history of diuretic use, according to the diagnostic algorithm for hyponatremia based on the European guideline (Figure 1),^2^ the most likely explanation was adrenal insufficiency (AI). Therefore, lab tests to evaluate adrenal function were ordered. The results showed basal morning cortisol was 6.2 µg/dL (7‐28 µg/dL) and ACTH 125 pg/mL (9‐52 pg/mL), which suggests primary adrenal insufficiency. The patient failed her cortisol stimulation test afterward which confirmed the diagnosis. Thyroid function test showed TSH 12.1 mU/L (0.5‐5.0 mU/L)and free T4 0.7 ng/dL (0.9‐2.4 ng/dL) which revealed inadequately controlled hypothyroidism.

Review of the history: The patient had fatigue, poor appetite, unintentional weight loss, and tanned skin for at least 1 month. Five days before admission, which was 3 days after taking TMP‐SMX, the patient started to have nausea and worsening fatigue. Based on the whole history and test results, our impression was that she had baseline mild‐to‐moderate chronic hyponatremia due to uncontrolled primary adrenal insufficiency, and this situation was acutely exacerbated by TMP‐SMX use several days prior. Obviously, the cause of the patient's hyponatremia was multifactorial. We also believe that inadequately controlled hypothyroidism due to self‐switching from levothyroxine to animal thyroid extract a few months prior may have played a minor role in the development of the hyponatremia.

### 2.4 Treatment

**Figure 2:**
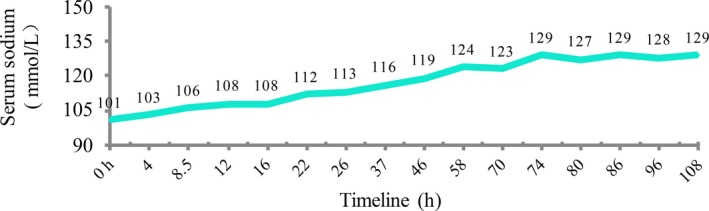
Serum sodium level during hospitalization

After the initial diagnosis of severe hyponatremia, the TMP‐SMX was discontinued immediately. The patient was given normal saline 60 mL/h, combined with fluid restriction limiting water intake to 1.5 L per day. The patient was given neither hypertonic saline nor tolvaptan because she had chronic hyponatremia and was neurologically asymptomatic. According to the guidelines, the patient has risk factor for osmotic demyelination syndrome (ODS) (Serum Na < 105 mmol/L), optimal correction rate should be <8 mmol/L per day. The patient's serum sodium level increased from 101 to 112 mmol/L in the first 22 hours, so we drove the correction rates down by holding the normal saline infusion. The patient's sodium level increased 5‐8 mmol/L per day in the following days which was at our goal. The patient was monitored closely in the process of hyponatremia correction. No neurologic symptoms or signs of ODS were observed. Her serum sodium had increased gradually to 129 mmol/L with significant clinical improvement in the following days (Figure 2).

Based on the final diagnosis of primary AI, the patient was treated with prednisone 5 mg daily and fludrocortisone 0.1 mg daily. At the same time, she was started on levothyroxine instead of thyroid extract to better control her hypothyroidism. The patient was discharged home with outpatient follow‐up. Sodium level was 129 mmol/L at discharge.

## 3 DISCUSSION

Hyponatremia is one of the most common electrolyte disorders in clinical practice. It is defined as a serum sodium concentration < 135 mmol/L and is considered severe when the serum level < 125 mmol/L. Symptoms of hyponatremia are primarily neurologic, ranging from nausea, vomiting, headache and malaise, to altered mental status, seizures, even coma, brain‐stem herniation, and respiratory arrest, which can lead to permanent brain damage or death. In our patient, her sodium level was 101 mmol/L on admission, but surprisingly, the patient was neurologically asymptomatic. Such a low level of serum sodium without any CNS symptoms has not been reported before.

The severity of hyponatremia depends on the rate and extent of changes in sodium concentration. In chronic hyponatremia, neurologic symptoms are much less severe due to cerebral adaption which begins within a day of sustained hyponatremia and generally takes several days for full measures to be in place. The adaptive mechanism involves (a) a compensatory displacement of fluid from the interstitial space into the cerebrospinal fluid and from there into the systemic circulation and (b) the extrusion of intracellular solutes together with osmotically obligated water to reduce cellular swelling and normalize brain volume. The process of brain volume regulation is complex and essential to understand the variability of the clinical presentation of hyponatremia.

Severe hyponatremia is associated with high morbidity and mortality. In order to implement correct treatment, an accurate clinical assessment must be made, focusing on fluid status, chronicity, and potential etiology. As in our patient, a majority of hyponatremia patients had a complex and multifactorial etiology. It is crucial that a thorough investigation is made to secure the correct diagnosis.

### 3.1 Hyponatremia and adrenal insufficiency

Hyponatremia is one of the major electrolyte abnormalities of primary adrenal insufficiency. It is mediated by increased release of antidiuretic hormone (ADH) which results in water retention and a reduction in the plasma sodium concentration.^3^, ^4^ The possible mechanisms may include the following: Cortisol directly suppressing ADH secretion, the removal of the inhibitory effect causes ADH hypersecretion,^5^, ^6^, ^7^, ^8^, ^9^ cortisol deficiency results in increased hypothalamic secretion of corticotropin‐releasing hormone (CRH), which is an ADH secretagogue 5, 10, 11 and renal salt elimination with resultant volume depletion and blood pressure reduction in AI cause hypersecretion of ADH. Several studies have revealed hypocortisolism may increase renal sensitivity to ADH, evidenced by upregulation of aquaporin‐2 water channel in glucocorticoid‐deficient rats.^12^, ^13^

Primary adrenal insufficiency may well be recognized by clear hallmarks of the disease, such as hyponatremia, hyperkalemia, hypotension, hyperpigmentation, and salt craving. It is an important diagnosis not to be missed since the consequences could be fatal.

### 3.2 Trimethoprim‐sulfamethoxazole‐induced hyponatremia

TMP‐SMX is a commonly prescribed antibiotic for various infections. TMP is structurally related to the potassium‐sparing diuretic amiloride and has been associated with hyperkalemia and hyponatremia through blocking epithelial sodium channels in the collection duct.

Electrolyte disorders are the most common adverse events in HIV‐infected patients treated with high doses of TMP‐SMX, while hyperkalemia is much more common than hyponatremia, occurring in as many as 44%‐70% of HIV patients treated with high‐dose TMP‐SMX.^14^, ^15^, ^16^ However, a recent single‐center retrospective study showed a high incidence (72.3%) of hyponatremia associated with the use of high‐dose TMP‐SMX (8 mg/kg/d of TMP for ≥3 days) among hospitalized patients, and hyponatremia was noted on average 5.5 days after initiation of therapy.^17^ Besides, a few reports have shown that even standard dose TMP‐SMX can cause hyponatremia in non‐HIV infected patients as well.^18^, ^19^, ^20^, ^21^ A retrospective study confirmed that TMP‐SMX is implicated not only in hyperkalemia but also in hyponatremia. These electrolyte disorders were found in 14 of 53 patients (26.4%), the average dose of TMP was 145.7 ± 24.9 mg/d, which was not high. When the patients were monitored carefully, electrolyte disorders were seen even in the low‐dose group. The incidence of electrolyte disorders is increased with the dose of TMP, indicating a dose‐dependent manner.^18^

In our case report, since the patient took standard dose TMP‐SMX for sinusitis prior to onset of symptoms, we believe TMP‐SMX is the acute aggravating factor which contributes to the ultimate profound hyponatremia. TMP blocks sodium reabsorption at epithelial sodium channel in the distal nephron, leading to hyperkalemia, and inevitably, hyponatremia as a result. There are also animal studies showed TMP infusion decreases renal potassium excretion by 40% and increases renal sodium excretion by 46%, likely in a dose‐dependent manner.^22^ Although standard dose TMP‐SMX‐induced hyponatremia is rare, it is still worth close attention in clinical practice.

The association between hyponatremia and TMP‐SMX emphasizes the need to evaluate electrolytes in patients who develop illness shortly after taking this medication, especially when patient has other risk factors or predisposing conditions of hyponatremia at the same time.

### 3.3 Hyponatremia and hypothyroidism

Hyponatremia has been reported in patients with moderate‐to‐severe hypothyroidism, particularly in patients with myxedema. This is due in part to a reduced cardiac output, which can lead to the release of ADH via the carotid sinus baroreceptors.^23^, ^24^ On the other hand, the glomerular filtration rate has been reported to be decreased in hypothyroidism, which leads to diminished free water excretion and dilutional hyponatremia.^25^, ^26^ Warner et al^27^ described a statistically significant association between hyponatremia and hypothyroidism: for every 10 mIu/L rise in thyroid‐stimulating hormone, serum sodium decreased 0.14 mmol/L. Our patient has moderate hypothyroidism with elevated TSH of 12.1 mIU/L, which is considered a small portion if any which may contribute to the development of hyponatremia.

### 3.4 Optimal correction rates of hyponatremia

Overly rapid correction of severe, chronic hyponatremia (serum sodium concentration <120 mmol/L and particularly <115 mmol/L) can lead to a severe and sometimes irreversible neurologic disorder, osmotic demyelination syndrome (ODS).^28^, ^29^ According to United States and European hyponatremia guidelines,^2^ the limit of correction rates in hyponatremia should be around 10 mmol/L per day for both acute and chronic hyponatremia.^2^ Of note, the United States guideline recommends a lower limit of 8 mmol/L per day in patients with high risk of ODS (sodium ≤ 105 mmol/L, concurrent hypokalemia, alcoholism, malnutrition, or hepatic disease). Many physicians advocate a more conservative rate of correction of 6 mmol/L per day,^28^, ^30^ although this is likely to be both sufficient and safe, the data to support this are still limited. During treatment, close monitoring of serum sodium is essential and prompt action should be taken to avoid overly rapid correction.

### 3.5 Treatment of hypothyroidism

Switching to animal thyroid extract by the patient is considered the contributor to the inadequate treatment of hypothyroidism. Regarding the treatment of hypothyroidism,^31^ the guidelines for the treatment of hypothyroidism by American Thyroid Association recommend levothyroxine as routine care for patients with primary hypothyroidism, in preference to the use of thyroid extracts. It is because of potential safety concerns related to the use of thyroid extracts, such as the presence of supraphysiologic serum triiodothyronine levels and a paucity of long‐term safety outcome data.

## 4 CONCLUSION

Although hyponatremia is common in clinical practice, our case report highlights that: (1) Profound hyponatremia with sodium level 101 mmol/L could have no CNS symptoms due to the remarkable adaption of brain; (2) severe hyponatremia is often multifactorial: (a) Even standard dose TMP‐SMX is associated with hyponatremia which emphasized the need for greater awareness of this serious and potentially fatal complication. (b) Endocrine disorders including adrenal and thyroid dysfunction should be considered in the differential diagnosis of hyponatremia and part of hyponatremic workup. (c) Thorough evaluation is required to uncover all the predisposing conditions, and each of them must be addressed to correct hyponatremia.

## CONFLICT OF INTEREST

None declared.

## AUTHOR CONTRIBUTIONS

NZ: conceived the idea, wrote the initial draft and edited the final manuscript. CY: supervised and revised the manuscript.
